# Prenatal diagnosis in Sweden 2011 to 2013—a register-based study

**DOI:** 10.1186/s12884-016-1165-8

**Published:** 2016-11-22

**Authors:** Kerstin Petersson, Marie Lindkvist, Margareta Persson, Peter Conner, Annika Åhman, Ingrid Mogren

**Affiliations:** 1 Department of Clinical Sciences, Obstetrics and Gynecology, Umeå University, Umeå, Sweden; 2 Department of Statistics, USBE; Department of Public Health and Clinical Medicine, Epidemiology and Global Health, Umeå University, Umeå, Sweden; 3 Department of Nursing, Umeå University, Umeå, Sweden; 4 Department of Obstetrics and Gynecology, Center for Fetal Medicine, Karolinska University Hospital, Stockholm, Sweden

**Keywords:** Pregnant women, Prenatal diagnosis, Uptake, Guidelines, Antenatal Care

## Abstract

**Background:**

Prenatal diagnosis involves methods used in early pregnancy as either screening tests or diagnostic methods. The aims of the study were to *i*) investigate guidelines on prenatal diagnosis in the counties of Sweden, *ii*) investigate uptake of prenatal diagnosis, and *iii*) background characteristics and pregnancy outcomes in relation to different prenatal diagnostic methods.

**Methods:**

A retrospective cross-sectional study using data from the Swedish Pregnancy Register 2011 to 2013 (284,789 pregnancies) was performed. Additionally, guidelines on prenatal diagnosis were collected. Biostatistical and epidemiological analyses were performed including calculation of odds ratios (OR) and their 95% confidence intervals in univariate and multivariate logistic regression analyses.

**Results:**

The national uptake of routine ultrasound examination, Combined Ultrasound and Biochemical test (CUB), Amniocentesis (AC) and Chorionic Villus Sampling (CVS) were 97.6, 33.0, 2.6 and 1.1%, respectively. From 2012, 6/21 counties offered CUB test to all pregnant women, nine counties at specific indications, and five counties did not offer CUB at all. Advanced maternal age demonstrated the highest impact on uptake of prenatal diagnosis. Further, university educational level in relation to lower educational level was associated with an increased likelihood of undergoing CUB (OR 2.30, 95% CI 2.26–2.35), AC (OR 1.54, 95% CI 1.46–1.63) and CVS (OR 2.68, 95% CI 2.44–2.93).

**Conclusion:**

Offers of prenatal diagnosis varied considerably between counties resulting in unequal access to prenatal diagnosis for pregnant women. The intentions of the Swedish Health and Medical Services Act stating equal care for all, was thus not fulfilled.

## Background

Antenatal care (ANC) is free of charge which almost all pregnant women in Sweden attend [1]. Most pregnant women are managed by public ANC facilities but private ANC clinics are also available. Sweden is divided into 21 counties, including 43 maternal health care areas (MHCAs). The 43 MHCAs issue medical guidelines based on national recommendations, local health care organization, and local policy related to surveillance of pregnancy [1]. For each MHCA, an antenatal care obstetrician and an antenatal care coordinator (midwife) are responsible for the medical guidelines. ANC in Sweden is mainly organized within the primary health system, but exceptionally it is integrated within hospital systems. Midwives working in ANC units are responsible for the monitoring of pregnant women with regard to current medical guidelines, and are responsible of referral of patients to hospital clinics when indicated. Information on prenatal diagnosis is provided by midwives in ANC, whereas prenatal screening or diagnostic procedures are generally undertaken in hospital-based clinics. In addition, midwives in ANC have to manage different administrative systems related to provision of health care, such as keeping medical records, and entering data into the Swedish Pregnancy Register.

### The Maternal Health Care Register and the Swedish Pregnancy Register

The Swedish Maternal Health Care Register (MHCR) is a national quality register where pregnant women participate on a voluntary basis by contributing with information on their pregnancy and delivery [2]. MHCR has collected data on outcomes of pregnancy, delivery, and the postpartum period since 1999. The quality of data recorded in the MHCR has previously been investigated, and show that most variables in the MHCR demonstrated good to a very good degree of coverage of data, and satisfying internal validity [3]. The MHCR was integrated in the Swedish Pregnancy Register (SPR) in January 1st 2013, in a merge of three registers involved in the different aspects of health care during pregnancy. The proportion of pregnancies registered in the MHCR was 81 and 85% during 2011 and 2012. The participation rate in the SPR during 2013 was further increased, reaching 89% of all women continuing pregnancy.

The National Board of Health and Welfare in Sweden has issued regulations concerning counselling on different methods of early prenatal screening and diagnostic procedures [4]. These regulations state that pregnant women and their partners must be offered this information at first visit in ANC. Early prenatal diagnosis is defined as prenatal screening and diagnostic procedures during the first 22 weeks of gestation [5]. Prenatal screening or diagnostic procedures during the first 22 weeks of gestation include the second trimester scan usually performed at a gestational age of 18 to 20 weeks, Combined Ultrasound and Biochemical test (CUB) and invasive tests such as either Chorionic Villus Sampling (CVS) or Amniocentesis (AC). CUB is a screening test, basically used to estimate the risks of trisomy 13, 18, and 21. The CUB test is performed during the first trimester when maternal serum samples are collected followed by a nuchal translucency scan during the gestational period of 11 to 13 + 6 days [6, 7]. The combined likelihood ratios are then calculated, and when the risk of Down’s syndrome is estimated to be higher than 1/200 above, the woman is offered an invasive procedure in order to obtain a certain diagnosis [8]. An invasive test may be a consequence of CUB, but may also be performed due to a known or suspected genetic condition that may be determined by DNA-PCR, CGH array or specific mutation analysis [5]. AC may be performed following 15 completed weeks, due to the increased risk of miscarriage or clubfoot, if the procedure is performed at an earlier gestational age [9].

The rationale of this study was to investigate the utilisation of prenatal diagnosis in Sweden during the study period 2011 to 2013 in relation to the different offers of prenatal screening and diagnostic procedures on a national level and comparing different counties.

### Aims

The overall aim was to investigate background characteristics and pregnancy outcomes in relation to the use of prenatal screening methods and diagnostic procedures in Sweden.

The specific aims of the study were to *i*) investigate guidelines on prenatal diagnosis in the counties of Sweden, *ii*) investigate uptake of routine ultrasound examination, combined ultrasound and biochemical test (CUB), chorionic villus sampling (CVS) and amniocentesis (AC), and *iii*) background characteristics and pregnancy outcomes in relation to different prenatal screening and diagnostic procedures.

## Methods

### Study design and setting

This retrospective, cross-sectional, epidemiological study analysed data on pregnancies from the Swedish Maternal Health Care Register and the Swedish Pregnancy Register from 2011 to 2013. MHCR was an independent register until 2012. MHCR was integrated into the SPR, as one of three registers when the SPR was formed in 2013. Here, SPR refers to MHCR and SPR as one entity. Inclusion criteria, for participating in the study, were being a subject included in the SPR with a date of delivery of a live or stillborn child from January 1st 2011 to December 31st 2013, and with a gestational age of 22 weeks and 0 days to 43 weeks and 0 days. Data on all pregnancies 2011 to 2013 were obtained from the SPR, comprising 284,789 women and their offspring. The participation rate of pregnant women in SPR was during 2011, 2012, and 2013, 81, 85 and 89%, respectively. The coverage of variables in relation to county, varied from 74 to 99% during 2013. Additionally, medical guidelines regarding offers to pregnant women on prenatal screening and diagnostic procedures were collected from each Maternal Health Care Area (MHCA; *N* = 43) in Sweden for 2011, 2012 and 2013. The guidelines during this study period were almost consistent with the exception of changes in two counties where no pregnant women previously had been offered CUB until 2011. A new guideline was introduced during 2012 in these two counties, offering all pregnant women CUB. The proportion of births in these two counties corresponds to 4% of all births in Sweden. Sweden includes 21 different counties where the majority of counties host only one MHCA, whereas some larger cities host multiple MHCAs, as for example the area of the capital Stockholm. Results related to guidelines will be presented on county-level.

### Definitions of variables

Some variables acted both as independent and dependent variables in analyses. See the descriptions below.

#### Independent variables

*Maternal age* was defined as age in years at delivery. *Parity* was defined as total number of children born (including the index pregnancy in the SPR). *Primiparity* was defined as having delivered one child, i.e. including the index pregnancy, and *multiparity* was defined as having delivered at least 2 children (in two pregnancies or more, including the index pregnancy). *Body mass index* (BMI) was calculated with the formula BMI = kg/m^2^. The different BMI groups were defined in accordance with the WHO’s definition of BMI: underweight: <18.50 kg/m^2^; normal weight: 18.50–24.99 kg/m^2^; overweight: 25.00–29.99 kg/m^2^, obesity class 1: 30.00–34.99 kg/m^2^, obesity class 2: 35.00–39.99 kg/m^2^, and obesity class 3: ≥40.00 kg/m^2^ [10]. *Level of education* was defined as elementary school, high school or university. *Employment status* was categorized into “employed”, “student”, “parental leave”, “unemployed”, “sick leave”, and “other status”. *Country of origin* was categorized into Sweden, other Nordic countries (Norway, Finland, Iceland, and Denmark) and Europe (excluding Sweden and other Nordic countries), Africa, Asia and other countries. The variable *Alcohol screening* (Alcohol Use Disorder Identification Test = AUDIT scores) was categorized in whether performed “yes” or “no”. *AUDIT-score* ranged from 0 to 40 scores, and was categorized into ≤5 scores and ≥6 scores, which indicates harmful alcohol use [11]. *Self-rated health prior to pregnancy* was categorized in “very good”, “good”, “neither good nor poor”, “poor”, and “very poor”. The following variables were categorized in “yes” or “no”: *smoking at 3 months prior to pregnancy*, *smoking at first ANC visit*, *smoking at 32 weeks of gestation*, *use of snuff 3 months prior to pregnancy*, *use of snuff 3 months at first ANC*, *use of snuff at 32 weeks of gestation*, *counselling due to fear of childbirth* where fear of childbirth was defined in the SPR as a subject being referred for counselling due to fear of childbirth, *treatment of psychiatric disorder* where psychiatric disorder was defined in the SPR as either medical or psychological treatment of psychiatric disorder, or both, *combined ultrasound and biochemical test* (CUB), *chorionic villus sampling* (CVS) and *amniocentesis* (AC).

#### Dependent variables

The following variables were categorized into “yes” or “no”: *combined ultrasound and biochemical test* (CUB), *chorionic villus sampling* (CVS), *amniocentesis* (AC), *counselling due to fear of childbirth* and *treatment of psychiatric disorder. Gestational age* was reported in days of gestation and presented as a continuous variable. *Mode of delivery* was categorized in “vaginal delivery”, “instrumental delivery” (including delivery with vacuum extraction or forceps), and “caesarean section”. Caesarean section (CS) was further categorized in “elective caesarean section” and “emergency caesarean section”. *Birth weight* in grams was presented as a continuous variable.

### Statistics

Categorical variables were analysed with frequencies and percentages. Continuous variables were presented by their mean value and standard deviation (SD), and by their median value and interquartile range (IQR). Continuous variables were tested for the assumption of normal distribution. Test of trend was analysed by Linear-by-Linear Association for investigation of linear trends over the years. Test of difference between independent groups were analysed with One-Way Anova test and independent samples t-test for parametric data, corrected for homogeneity for variance if necessary. The Pearson’s Chi-Square test was used for test of difference between groups for categorical data. Level of significance was set at *p* < 0.05. Odds ratios (OR) and their 95% confidence intervals (CI) were calculated in univariate and multivariable logistic regression analyses. SPSS vs. 22 and vs. 23 were used for these calculations. A Venn diagram was created to present the uptake of CUB, CVS and AC in the study sample. A figure presenting a map of Sweden was created to illustrate geographical differences in uptake of CUB, where the 21 counties were categorized into 4 groups of CUB uptake rate: less than 10%, 10 to 29.99%, 30 to 69.99% or 70% or more.

## Results

### Offers on prenatal diagnosis in Sweden

**Fig. 1:**
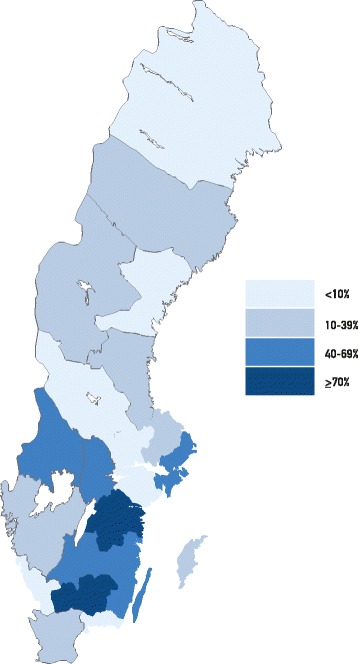
Map of Sweden presenting the 21 counties and their uptake of CUB categorised into four levels

All counties in Sweden except one had issued written guidelines concerning offers of prenatal screening methods and diagnostic procedures during the first and second trimesters of pregnancy. These guidelines remained unchanged in all counties except for two counties, during the study period. Three Swedish counties offered a routine ultrasound examination at 12 weeks of gestation for the purpose of dating and all other counties offered a second trimester scan at the gestational age of 17–20 weeks, with the exception of one county that accepted dating from week 16. CUB was offered to all pregnant women in six counties, was offered on indication advanced maternal age in nine counties, and was not offered at all in five counties. The definition of advanced maternal age as indication for CUB demonstrated a substantial variation between counties. The different cut-off-values defining an indication for offering the CUB test were: age >33 years at last menstrual period, age ≥35 years at last menstrual period, age ≥35 years at conception, age ≥35 years at the time for the CUB-test, and age ≥35 years at the estimated date of delivery. In addition, one county included “anxiety related to pregnancy” as an indication for offering CUB. All counties offered either CVS or AC as prenatal diagnostic procedures on the indications: maternal age ≥35 years, increased risk for chromosomal aberration following CUB, or second trimester serum screening, or familial genetic condition. Three counties offered AC only if CUB had previously been performed and indicated an increased risk. During 2013, the uptake of CUB varied between the counties from 2.2% to 80.3%, (Table 1). Figure 1 shows a map of Sweden presenting the 21 counties and their uptake of CUB categorized into four levels. The lowest uptake rate, i.e. uptake less than 10%, corresponds to counties where no pregnant women were offered CUB. Pregnant women, living in any of the counties where no women were offered CUB, could still have undergone CUB but if so, privately and at their own expense.

**Table 1:** Uptake of Combined Ultrasound and Biochemical test (CUB) per County (*N* = 21) 2011 to 2013

County	CUB			Proportions^a^
	2011	2012^b^	2013	2013
	%	%	%	%
1	41.0	47.4	53.2	26.1
2	18.4	19.5	21.4	17.0
3	26.4	27.8	30.1	13.8
4	81.4	78.9	80.3	4.4
5	23.7	25.0	24.9	3.5
6	64.9	66.6	66.9	3.4
7	6.7	7.9	7.0	2.9
8	59.5	61.6	63.4	2.9
9	2.4	3.0	2.7	2.7
10	5.1	5.1	3.9	2.5
11	10.4	11.7	11.0	2.5
12	6.4	9.3	9.7	2.4
13	48.9	56.3	60.9	2.4
14	1.1	1.7	2.2	2.2
15	5.3	6.7	8.8	2.1
16	13.1	13.7	12.6	2.1
17	2.9	28.8	66.7	2.1
18	4.2	18.9	75.1	1.9
19	2.0	3.9	5.3	1.3
20	13.3	16.2	15.1	1.1
21	12.5	14.6	14.2	0.5
Total	29.8	32.5	36.2	100

^a^Proportions of births per county in relation to the total number of births in Sweden 2013 (*N* = 113,593)

^b^County no 17 and no 18 changed their guidelines of prenatal diagnosis during 2012

### The study population

**Fig. 2:**
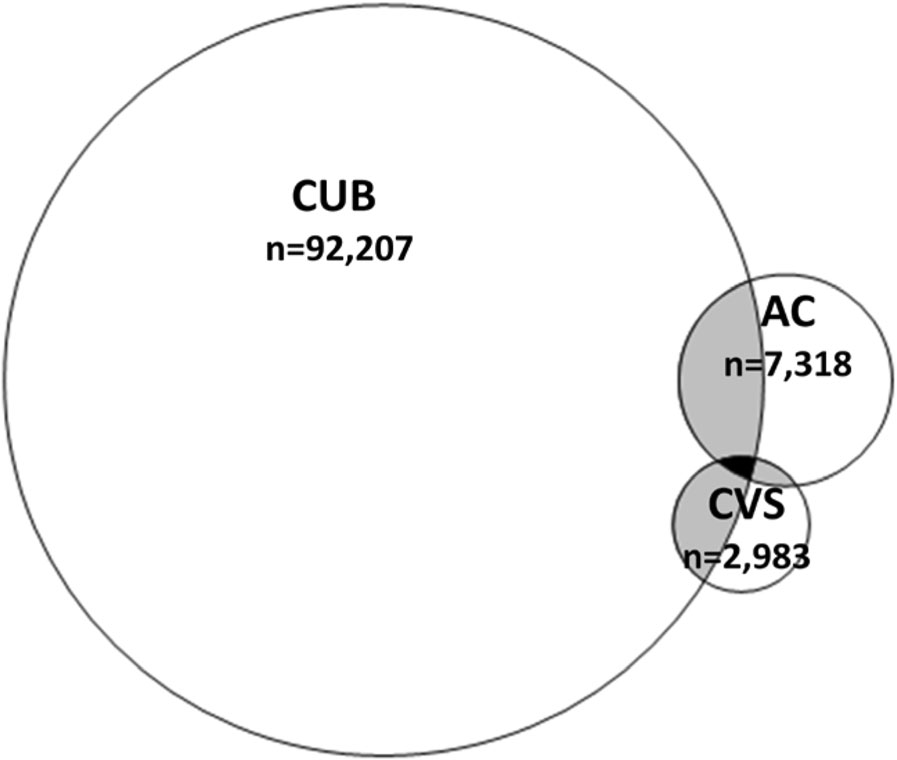
Pregnant women that were examined with CUB, AC and CVS

The study population included 284,789 pregnant women, and the distribution of participants per year was 30.9% (2011), 33.7% (2012) and 35.3% (2013). Background characteristics for the study population are presented in Table 2. Mean age and mean body mass index (BMI) were 30.24 years and 24.79 kg/m^2^, respectively. Mean age and body mass index of primiparous and multiparous women were 28.83 years and 31.72 years, and 24.34 kg/m^2^ and 25.14 kg/m^2^, respectively (Table 2). For multiparous women, the variable maternal age was normally distributed 2012 and 2013 (Table 2). For primiparous women who had undergone CUB-test the variable maternal age was normally distributed (Table 4). All other continuous variables demonstrated skewness to some degree. Almost all pregnant women were examined with a routine ultrasound scan during pregnancy (97.6%), and the overall proportions of women examined with CUB, CVS or AC were 33.0, 1.1 and 2.6% (Table 3). The percentage of women examined with CUB increased significantly during the study period from 29.8% in 2011 to 36.2% in 2013 (*p* < 0.001) (Table 3). The number of pregnant women, who were examined with CUB, CVS or AC, or any combinations of these procedures, was in total 98,697, which corresponds to 33.4% of all women and is presented in a Venn diagram (Fig. 2). Of all pregnant women who were examined with CUB, 1.1% (*n* = 1252) thereafter underwent CVS, and 2.7% (*n* = 2493) underwent AC after CUB. Of all women who were examined with CVS (*n* = 2970), 42.2% had undergone CUB prior to CVS. Of all women who were examined with AC (*n* = 7279), 34.2% had undergone CUB prior to AC.

**Table 2:** Background characteristics and pregnancy outcomes in the Swedish Pregnancy Register 2011 to 2013 (*N* = 284,789)

Variable	Total		2011		2012		2013		Test of difference^a^
	*N* = 284,789		*n* = 88,140		*n* = 96,043		*n* = 100,606		
	n	%	n	%	n	%	n	%	
Maternal age^b^, primiparous women (years)									
Mean (SD)^c^	28.83 (5.12)		28.78 (5.17)		28.82 (5.14)		28.87 (5.07)		0.053
Min-max	13.49–56.30		13.52–52.64		13.78–56.30		13.49–52.89		
Median (IQR)^d^	28.64 (7.07)		28.66 (7.08)		28.59 (7.05)		28.67 (7.04)		
Maternal age^b^, multiparous women (years)									
Mean (SD)^c^	31.72 (4.90)		32.29 (4.89)		32.21 (4.90)		32.16 (4.89)		<0.001
Min-max	15.00–57.00		17.07–53.25		16.41–54.47		15.67–57.34		
Median (IQR)^d^	32.30 (6.88)		32.41 (6.91)		32.28 (6.84)		32.23 (6.89)		
Maternal age^b^ in age-groups (years)									
< 20	3976	1.4	1369	1.6	1319	1.4	1288	1.3	
20–24	38,832	13.6	11,990	13.6	13,222	13.8	13,611	13.5	
25–29	84,129	29.5	25,578	29.0	28,393	29.6	30,158	30.0	
30–34	95,772	33.6	29,463	33.4	32,243	33.6	34,066	33.9	
35–39	50,717	17.8	16,215	18.4	17,010	17.7	17,492	17.4	
40–44	10,705	3.8	3323	3.8	3625	3.8	3757	3.7	
> 44	583	0.2	167	0.2	213	0.2	203	0.2	
Body mass index (kg/m^2^)									
Mean (SD)^c^	24.79 (4.65)		24.75 (4.62)		24.82 (4.67)		24.81 (4.66)		0.002
Min-max	13.03–71.63		13.82–62.06		13.63–67.22		13.03–71.63		
Median (IQR)^d^	23.80 (5.00)		23.74 (5.41)		23.81 (5.45)		23.81 (5.52)		
< 18.5	6838	2.5	2044	2.4	2328	2.5	2466	2.5	
18.5–24.99	163,856	59.2	50,787	59.7	55,203	58.9	57,866	58.9	
25–29.99	70,440	25.4	21,478	25.3	23,870	25.5	25,092	25.5	
30–34.99	25,166	9.1	7526	8.8	8573	9.2	9067	9.2	
35.39.99	7899	2.9	2374	2.8	2734	2.9	2791	2.8	
≥ 40	2759	1.0	836	1.0	969	1.0	954	1.0	
Educational level									
Elementary school	20,860	8.7	6662	9.4	6870	8.5	7328	8.4	<0.001
High school	95,564	40.0	27,821	39.4	32,660	40.3	35,083	40.1	
University	122,623	51.3	36,155	51.2	41,432	51.2	45,036	51.5	
Main occupation									
Employed	195,880	70.3	59,890	70.3	66,622	70.5	69,368	70.1	<0.001
Student	31,021	11.1	9697	11.4	10,364	11.0	10,960	11.1	
Parental leave	20,469	7.3	6082	7.1	6911	7.3	7475	7.5	
Unemployed	15,163	5.4	4955	5.8	4996	5.3	5212	5.3	
Sick leave	4421	1.6	1241	1.5	1557	1.6	1623	1.6	
Other	11,686	4.2	3300	3.9	4013	4.2	4373	4.4	
Country of birth									
Sweden	221,398	79.4	70,376	81.8	74,187	78.9	76,835	77.8	<0.001
Other Nordic countries^e^	2374	0.8	613	0.8	832	0.8	929	0.8	
Europe^f^	12,861	4.6	3486	4.0	4481	4.8	4900	5.0	
Africa	10,782	3.9	2780	3.2	3658	3.9	4344	4.4	
Asia	25,597	9.2	6886	8.0	8976	9.5	9735	9.9	
Other	5902	2.1	1917	2.2	1940	2.1	2045	2.1	
Smoking 3 months prior to pregnancy	38,854	13.8	12,134	13.9	13,285	14.0	13,435	13.5	0.004
Smoking at first ANC^g^ visit	15,874	5.6	5062	5.8	5475	5.7	5337	5.4	<0.001
Smoking at 32 weeks of gestation	11,990	4.2	3923	4.5	4133	4.3	3934	3.9	<0.001
Use of snuff 3 months prior to pregnancy	9954	3.5	2530	2.9	3506	3.7	3918	3.9	<0.001
Use of snuff at first ANC^g^ visit	2858	1.0	793	0.9	945	1.0	1120	1.1	<0.001
Use of snuff at 32 weeks of gestation	1721	0.6	548	0.6	561	0.6	612	0.6	0.572
Alcohol screening (AUDIT)^h^	245,544	88.1	74,712	86.7	81,408	86.7	89,424	90.6	<0.001
AUDIT-score^j^									
Mean (SD)^c^	2.19 (2.19)		2.28 (2.22)		2.20 (2.22)		2.09 (2.15)		
Min-max	0–40		0–39		0–40		0–40		
Median (IQR)^d^	2.00 (2)		2.00 (2)		2.00 (2)		2.00 (3)		
≤ 5p	229,854	94.0	69,110	93.6	76,327	93.8	84,417	94.4	<0.001
≥ 6p	14,751	6.0	4704	6.4	5044	6.2	5003	5.6	
Self-rated health prior to pregnancy	241,854	84.9	70,633	80.1	82,594	86.0	88,627	88.1	
Very good	72,321	29.9	19,838	28.1	24,472	29.6	28,011	31.6	<0.001
Good	141,251	58.4	41,299	58.5	48,441	58.6	51,511	58.1	
Neither good nor poor	20,425	8.4	6793	9.6	6962	8.4	6670	7.5	
Poor	6325	2.6	2174	3.1	2197	2.7	1954	2.2	
Very poor	1532	0.6	529	0.7	522	0.6	481	0.5	
Counselling due to fear of childbirth	21,595	7.6	6518	7.5	7186	7.5	7891	7.9	0.001
Treatment of psychiatric disorder	17,724	6.3	5122	5.9	6061	6.4	6541	6.5	<0.001
Gestational age (days)									
Mean (SD)^c^	278.0 (13.8)		278.0 (13.9)		277.8 (13.9)		278.1 (13.7)		<0.001
Min-max	154–301		155–301		154–301		154–301		
Median (IQR)^d^	280.00 (13.00)		280.00 (13.00)		280.00 (13.00)		280.00 (13.00)		
Mode of delivery	283,660	99.6	87,915	99.7	95,594	99.5	100,151	99.5	
Vaginal	217,898	76.8	67,277	76.5	73,333	76.7	77,288	77.2	<0.001
Instrumental	19,177	6.8	6208	7.1	6616	6.9	6353	6.3	
Caesarean section	46,585	16.4	14,430	16.4	15,645	16.4	16,510	16.5	
Caesarean section (CS)									
Elective CS^j^	20,272	43.6	6321	43.9	6718	43.0	7233	43.9	0.193
Emergency CS^j^	26,214	56.4	8085	56.1	8897	57.0	9232	56.1	
Birth weight (grams)^k^									
Mean (SD)^c^	3542 (556)		3540 (557)		3543 (556)		3543 (556)		0.352
Min-max	300–6640		300–6050		305–6270		300–6640		
Median (IQR)^d^	3550 (670)		3550 (675)		3550 (674)		3550 (670)		

^a^Test of difference between years using One-Way Anova test on numeric variables, and Pearsons’s Chi-Square test for categorical variables

^b^Maternal age at delivery

^c^SD = Standard Deviation

^d^IQR = Interquartile Range

^e^Other Nordic countries includes Norway, Finland, Iceland and Denmark

^f^The Nordic countries are excluded

^g^Antenatal care

^h^Assessment of use of alcohol prior to pregnancy with screening instrument Alcohol Use Disorder Identification Test (AUDIT)

^i^ AUDIT score ranging from 0 to 40

^j^Caesarean section

^k^Singletons exclusively included in analysis

**Table 3:** Uptake of routine ultrasound examination, Combined Ultrasound and Biochemical test (CUB), Chorionic Villus Sampling (CVS) and Amniocentesis (AC) during 2011 to 2013, and test of trend^a^

Variable	Total		2011		2012		2013		Trend^a^
	*N* = 284,789		*n* = 88,140		*n* = 96,043		*n* = 100,606		
	n	%	n	%	n	%	n	%	
Ultrasound	281,562	98.9	85,561	97.1	97,500	99.6	100,310	99.7	
Yes	274,899	97.6	83,549	97.6	93,386	97.6	97,964	97.7	0.716
No	6663	2.4	2.012	2.4	2314	2.4	2337	2.3	
CUB	278,230	98.0	84,827	96.2	94,900	98.8	99,503	98.9	
Yes	92,207	33.0	25,316	29.8	30,826	32.5	36,065	36.2	<0.001
No	187,023	67.0	59,511	70.2	64,074	67.5	63,438	63.8	
CVS	280,898	98.6	85,308	96.8	95,465	99.4	100,125	99.5	
Yes	2983	1.1	868	1.0	927	1.0	1188	1.2	<0.001
No	277,915	98.9	84,440	99.0	94,538	98.8	98,937	98.8	
AC	280,667	98.6	85,213	99.3	95,395	99.3	100,059	99.5	
Yes	7318	2.6	2500	2.9	2473	2.6	2345	2.3	<0.001
No	273,349	97.4	82,713	97.1	92,922	97.4	97,714	97.7	

^a^Test of trend by Linear-by-Linear Association

Mean age for primiparous women who underwent CUB, CVS or AC was 31.34, 33.79 and 33.65 years, respectively (Table 4). Overall mean age for pregnant women who had not been examined with CUB, CVS or AC was 28.84 (defined as “all others” in Table 4), where the mean age for primiparous and multiparous women in this category was 27.09 and 30.29 years, respectively. BMI for pregnant women who had undergone CUB, CVS or AC was 24.40, 24.10 and 24.94, respectively, whereas BMI for pregnant women who had not undergone CUB, CVS or AC (“all others”) was 24.99. There was a statistically significant difference in BMI between those who underwent CUB and “all others” (*p*-value <0.001) (Table 4).

**Table 4:** Background characteristics and pregnancy outcomes in relation to prenatal screening or diagnostic procedures in the Swedish Pregnancy Register 2011 to 2013 (*N* = 284,789)

Variable	CUB^a^		CVS^b^		AC^c^		All others^d^		Test of difference^e^
	*n* = 92,207		*n* = 2983		*n* = 7318		*n* = 186,092		
	n	%	n	%	n	%	n	%	
Maternal age^f^, primiparous women (years)									
Mean (SD)^g^	31.34 (5.17)		33.79 (5.74)		33.65 (6.11)		27.09 (4.58)		<0.001
Min-max	15.09–56.30		17.29–49.03		16.74–49.83		13–54		
Median (IQR)^h^	31.34 (7.32)		34.41 (8.38)		34.92 (9.15)		27.51 (6.41)		
Maternal age^f^, multiparous women (years)									
Mean (SD)^g^	34.45 (4.45)		36.87 (4.65)		37.13 (4.66)		30.29 (4.52)		<0.001
Min-max	16.41–55.34		19.10–51.76		16.41–48.68		15–57		
Median (IQR)^h^	34.97 (5.72)		37.58 (5.66)		37.82 (5.45)		30.87 (6.07)		
Maternal age^f^ in age-groups (years)									
< 20	388	0.4	8	0.3	23	0.3	3566	1.9	
20–24	5863	6.4	99	3.3	313	4.3	32,702	17.6	
25–29	17,617	19.1	300	10.1	733	10.0	65,926	35.4	
30–34	31,792	34.5	649	21.8	1314	18.0	62,944	33.8	
35–39	30,179	32.7	1262	42.3	3148	43.0	17,676	9.5	
40–44	6093	6.6	628	21.1	1675	22.9	2991	1.6	
> 44	270	0.3	36	1.2	109	1.5	211	0.1	
Body mass index (kg/m2)									
Mean (SD)^g^	24.40 (4.34)		24.10 (4.10)		24.94 (4.52)		24.99 (4.80)		<0.001
Min-max	13.82–56.65		15.24–51.31		15.24–50.69		13.03–71.63		
Median (IQR)^h^	23.45 (5.0)		23.18 (5.0)		24.01 (5.0)		23.95 (6.0)		
< 18.5	1965	2.2	52	1.8	121	1.7	4766	2.6	
18.5–24.99	56,723	63.2	1918	66.3	4140	58.2	103,359	57.2	
25–29.99	21,639	24.1	663	22.9	1926	27.1	47,160	26.1	
30–34.99	6863	7.6	195	6.7	652	9.2	17,741	9.8	
35.39.99	1992	2.2	48	1.7	203	2.9	5744	3.2	
≥ 40	629	0.7	18	0.6	68	1.0	2071	1.1	
Educational level									
Elementary school	3242	4.1	69	2.7	334	5.3	17,353	11.2	<0.001
High school	24,324	31.1	608	23.7	2078	33.1	69,523	44.8	
University	50,763	64.8	1887	73.6	3869	61.6	68,302	44.0	
Main occupation									
Employed	72,519	80.3	2439	83.7	5582	77.8	118,371	65.0	<0.001
Student	6467	7.2	146	5.0	524	7.3	24,121	13.3	
Parental leave	5059	5.6	149	5.1	459	6.4	15,002	8.2	
Unemployed	3320	3.7	78	2.7	294	4.1	11,601	6.4	
Sick leave	1306	1.4	36	1.2	119	1.7	3009	1.7	
Other	1610	1.8	66	2.3	194	2.7	9897	5.4	
Country of birth									
Sweden	76,276	84.5	2482	85.4	5876	81.8	139,881	76.7	<0.001
Other Nordic countries^i^	904	1.0	31	1.1	74	1.0	1406	0.8	
Europe^j^	3971	4.4	102	3.5	332	4.6	8617	4.7	
Africa	1243	1.4	37	1.3	127	1.8	9438	5.2	
Asia	6132	6.8	196	6.7	590	8.2	18,955	10.4	
Other	1727	1.9	57	2.0	180	2.5	4007	2.2	
Smoking 3 months prior to pregnancy	9583	10.5	229	7.7	751	10.3	28,659	15.6	<0.001
Smoking at first ANC^k^ visit	3445	3.8	76	2.6	347	4.8	12,144	6.6	<0.001
Smoking at 32 weeks of gestation	2527	2.8	48	1.6	289	4.0	9238	5.0	<0.001
Use of snuff 3 month prior to pregnancy	2493	2.7	64	2.2	213	2.9	7268	3.9	<0.001
Use of snuff at first ANC visit	646	0.7	22	0.7	85	1.2	2134	1.2	<0.001
Use of snuff at 32 weeks of gestation	385	0.4	13	0.4	41	0.6	1303	0.7	<0.001
Alcohol screening (AUDIT)^l^	81,686	90.3	2568	88.4	6313	86.3	158,335	87.0	<0.001
AUDIT-score^m^									
Mean (SD)	2.34 (1.96		2.26 (1.74)		2.12 (1.90		2.11 (2.32)		
Min-max	0–38		0–18		0–29		0–40		
Median (IQR)^h^	2.00 (2)		2.00 (2)		2.00 (2)		2.00 (3)		
≤ 5p	76,786	94.5	2462	96.3	6012	95.7	82,067	94.6	<0.001
≥ 6p	4485	5.5	95	3.7	270	4.3	4703	5.4	
Self-rated health prior to pregnancy									
Very good	25,990	33.0	834	32.5	1887	30.5	44,643	28.3	<0.001
Good	44,563	56.6	1433	55.9	3489	56.5	93,611	59.4	
Neither good nor poor	5877	7.5	217	8.5	583	9.4	14,047	8.9	
Poor	1817	2.3	64	2.5	187	3.0	4334	2.7	
Very poor	440	0.6	15	0.6	34	0.6	1056	0.7	
Counselling due to fear of childbirth	8900	9.7	324	10.9	745	10.2	12,023	6.5	<0.001
Treatment of psychiatric disorder	6002	6.5	185	6.3	533	7.3	11,275	6.1	<0.001
Gestational age (days)									
Mean (SD)^g^	278.0 (13.7)		276.6 (15.0)		276.5 (15.4)		278.0 (13.8)		0.326
Min-max	154–301		158–300		157–301		154–301		
Median (IQR)^h^	280.0 (13.0)		279.0 (13.0)		279 (15.0)		280 (13.00)		
Mode of delivery									
Vaginal	6885	73.9	2079	70.2	5133	70.3	145,486	78.5	<0.001
Instrumental	6376	6.9	163	5.5	392	5.4	12,468	6.7	
Caesarean section	17,574	19.1	721	24.3	1772	24.3	27,405	14.8	
Caesarean section (CS) ^n^									
Elective CS^n^	8650	49.3	297	58.7	916	51.7	10,700	39.1	<0.001
Emergency CS^n^	8893	50.7	422	41.3	855	48.3	16,639	60.9	
Birth weight (grams)^o^									
Mean (SD)^g^	3549 (552)		3531 (585)		3510 (620)		3539 (557)		0.001
Min-max	310–6270		400–5710		370–5776		300–6640		
Median (IQR)^h^	3560 (665)		3555 (686)		3548 (706)		3545 (680)		

^a^CUB = Combined Ultrasound and Biochemical test

^b^CVS = Chorionic Villus Sampling

^c^AC = Amniocentesis

^d^All others = Pregnant women who did not undergo any of the prenatal diagnostic procedures CUB, CVS or AC

^e^Test of difference between the two groups; pregnant women who underwent CUB and “all others” using t-test for continuous variables and Pearson’s Chi-Square test for categorical variables

^f^Maternal age at delivery

^g^SD = Standard Deviation

^h^IQR = Interquartile Range

^i^ Other Nordic countries includes Norway, Finland, Iceland and Denmark

^j^ The Nordic countries excluded

^k^Antenatal care

^l^ Assessment of use of alcohol prior to pregnancy with screening instrument Alcohol Use Disorder Identification Test (AUDIT)

^m^AUDIT score range from 0 to 40

^n^Caesarean section

^o^Singletons exclusively included in analysis

Pregnant women who underwent CUB, CVS or AC reported being employed in significantly higher proportions (80.3, 87.0 and 77.8%, respectively), vs. 65% for women who had not undergone CUB, CVS or AC (*p*-value <0.001).

Information on country of birth was available for almost all participants (Table 2). Sweden as country of birth was reported by 79.4% of all pregnant women (Table 2). Pregnant women who had undergone CUB, CVS and AC, reported Sweden as country of birth in 84.5, 85.4 and 81.8% of the cases, respectively. The corresponding figure for pregnant women who had not been exposed to CUB, CVS or AC was 76.7%. That means that a significantly lower proportion of women who were born outside of Sweden was examined by CUB, CVS and AC (*p*-value <0.001) (Table 4).

A proportion of 35.2% of women with a Nordic origin were exposed to CUB. The corresponding figures for women born in Europe, Africa, and Asia were 31.5, 11.8 and 24.5% respectively. Smoking was reported to a significantly lower degree (*p*-value <0.001) at all three check points, i.e. 3 months prior to pregnancy, at first antenatal visit and at gestational age of 32 weeks, by women who had undergone CUB compared to those who had not been examined by CUB, CVS or AC, i.e. “all others” (Table 4).

Women who had been examined by CUB, CVS or AC reported having received counselling due to fear of childbirth in 9.7, 10.9 and 10.2% of cases, respectively. Women who had not been examined with CUB, CVS or AC reported having received counselling due to fear of childbirth in a significantly lower proportion (6.5%; *p*-value <0.001) (Table 4). There was no significantly difference between the group of women who had undergone CUB and women included in the group “all others” regarding gestational age at delivery (Table 4).

The overall proportion of CS in the study group was 16.4% (Table 2). For pregnant women who had undergone CUB, CVS and AC, the prevalence of CS were 19.1, 24.3 and 24.3%, respectively, and the corresponding figure for “all others” was significantly lower 14.8% (*p*-value <0.001) (Table 4).

If caesarean section had been performed, the proportions of those who underwent elective CS or emergency CS were as follows; women who had undergone CUB, 49.3% and 50.7%, women who had undergone AC, 51.7% and 48.3% and women who had undergone CVS, 58.7% and 41.3% respectively. The corresponding figures for women included in the group “all others” were 39.1% (elective CS) and 60.9% (emergency CS), (Table 4).

The odds ratio for undergoing CUB at a maternal age of 35 years or older, was highly increased (4.36; 95% CI 4.28–4.45). When the OR was adjusted for educational level the OR still remained increased (4.00; 95% CI 3.91–4.08). Table 5 presents univariate and multivariable logistic regression analyses for the uptake of CUB in relation to specific background characteristics. Educational level demonstrated a strong impact on the likelihood of being examined with a CUB test. Women under the age of 35 years, having attended university, had an Adjusted Odds Ratio (AOR) of 1.79 (95% Adjusted Confidence Interval (ACI) 1.75–1.83) for undergoing CUB (Table 5). The corresponding figure for women 35 years or older who had attended university was AOR 1.53 (95% ACI 1.47–1.61) (Table 5). Pregnant women with a BMI of 25 or more, women who were unemployed, women who were born outside of Sweden and women who reported ongoing smoking at their first visit at ANC demonstrated a decreased AOR for undergoing CUB (Table 5). Women under the age of 35 years who had received counselling due to fear of childbirth had an increased AOR of 1.38 (95% ACI 1.32–1.45) for undergoing CUB, whereas the corresponding figure for women 35 years or older was somewhat lower (1.27; 95% ACI 1.18–1.28). Women in both age groups, having received treatment for psychiatric disorder, demonstrated a small but statistically significant increased AOR for undergoing CUB (Table 5).

**Table 5:** Univariate and multivariable logistic regression analysis for undergoing Combined Ultrasound and Biochemical test (CUB) in relation to maternal age divided into two age groups and to specified background characteristics

	Maternal age <35 years				Maternal age ≥35 years			
Variable	Crude OR	CI 95%	Adjusted OR ^a^	Adjusted CI 95%	Crude OR	CI 95%	Adjusted OR^a^	Adjusted CI 95%
Educational level								
Elementary school, high school	1		1		1		1	
University level	2.03	1.98–2.07	1.79	1.75–1.83	1.86	1.79–1.93	1.53	1.47–1.61
Body mass index (kg/m^2^)								
< 25	1		1		1		1	
≥ 25	0.75	0.73–0.76	0.84	0.82–0.86	0.65	0.63–0.68	0.76	0.73–0.80
Main occupation								
Employed, student, parental leave	1		1		1		1	
Unemployed, sick leave, other	0.51	0.49–0.53	0.70	0.67–0.74	0.46	0.44–0.49	0.64	0.59–0.70
Country of birth								
Sweden	1		1		1		1	
Other	0.60	0.58–0.61	0.76	0.74–0.79	0.55	0.53–0.57	0.74	0.70–0.78
Smoking at first visit at antenatal care								
No	1		1		1		1	
Yes	0.62	0.59–0.65	0.87	0.82–0.92	0.59	0.54–0.64	0.74	0.66–0.84
Self-rated health prior to pregnancy								
Very good and good	1		1		1		1	
Poor and very poor	0.85	0.74–0.98	0.96	0.82–1.13	0.62	0.50–0.76	0.80	0.63–1.02
Counseling due to fear of childbirth								
No	1		1		1		1	
Yes	1.41	1.36–1.46	1.38	1.32–1.45	1.40	1.32–1.48	1.27	1.18–1.28
Treatment of psychiatric disorder								
No	1		1		1		1	
Yes	1.03	0.99–1.08	1.15	1.09–1.22	1.04	0.98–1.11	1.16	1.05–1.28

^a^Adjusted for all other variables included in the analysis

Maternal age demonstrated the highest impact on the likelihood of undergoing invasive prenatal diagnosis (AC: COR 7.97; 95% CI 7.58–8.38, and CVS: COR 6.72; 95% CI 6.23–7.24). Further, women who had achieved an educational level corresponding to university had an increased likelihood of undergoing AC (COR 1.54; 95% CI 1.46–1.62) and CVS (2.68; 95% CI 2.45–2.92), in relation to women with a lower educational level. Their increased odds ratios for AC and CVS remained unchanged after adjusting for maternal age (AC: AOR 1.54; 95% ACI 1.46–1.62, and CVS: AOR 2.68; 95% ACI 2.45–2.92). Additionally, pregnant women who had received counselling due to fear of childbirth demonstrated a higher likelihood of undergoing AC or CVS (AC: COR 1.39; 95% CI 1.29–1.50, and CVS: COR 1.49; 95% CI 1.32–1.67), in comparison to those who had not been counselled for fear of childbirth. When adjusted for age and educational level the likelihood remained significantly increased for AC (AOR 1.14; 95% ACI 1.04–1.24) and CVS (AOR 1.15; 95% ACI 1.01–1.31).

A decreased likelihood of undergoing AC or CVS was demonstrated for pregnant women who reported country of birth outside of Sweden, compared to those who were born in Sweden (AC: COR 0.85; 95% CI 0.80–0.91, and CVS: COR 0.66; 95% CI 0.59–0.73). When adjusted for age and educational level the odds ratios remained significantly decreased (AC: AOR 0.88; 95% ACI 0.82–0.95, and CVS: AOR 0.81; 95% ACI 0.72–0.91).

## Discussion

The aim of this study was to make a national survey on guidelines concerning offers on prenatal diagnosis in Sweden. Further, we aimed to investigate background characteristics and pregnancy outcomes in relation to the uptake of different prenatal diagnostic methods. During the study period of 2011 to 2013 in Sweden there was an absence of a national consensus regarding guidelines on offers of prenatal diagnosis. The Swedish law states that all pregnant women should be offered information on prenatal diagnosis [4]. However, the opportunities of undergoing different prenatal screening or diagnostic procedures were not equally distributed during the time period under study among Swedish counties. On a national level, the uptake of the second trimester scan, CVS and AC was relatively stable during the study period whereas the uptake of CUB increased from 29.8%, 2011 to 36.2% in 2013. A Danish study performed 2008, shows a sharp decline in the uptake of invasive prenatal diagnosis when implementing screening programs offering CUB [12]. A study exploring determinants of participating in the first trimester combined test shows that advanced maternal age is the primary indication and has the highest impact for uptake of CUB [13]. As expected, our study displayed increased maternal age as the factor with the highest impact on whether pregnant women were examined with a CUB test. Further, educational level and country of birth were also significant background factors for women’s utilization of CUB. A study investigating effects of knowledge, education and experience of first trimester screening shows that women with a university education have a higher degree of knowledge of first trimester screening [14]. A Swedish study from 2012, exploring the effects of a public video aiming for an informed choice in relation to exposure to second trimester ultrasound, shows that women with college or university educational level were more likely to make an informed choice [15]. In our study women who had achieved a university education were more likely to undergo the CUB test, and the effect of educational level was more pronounced for women younger than 35 years of age in comparison to women 35 years or older. It is likely that higher education implicates a higher ability to gain, interpret and use information on different health offers, health promotion or risk factors. Ethnicity in relation to uptake of prenatal diagnosis has been investigated in several previous studies [16–18]. A register-based study in the Netherlands shows that women with a North-African ethnic origin have the lowest participation rate in prenatal screening for Down’s syndrome, only 8% participation rate compared to the higher rate for women with a Dutch (28%) or other Western origin (33%) [16]. Also, an Australian study demonstrates that ethnicity is strongly associated with the uptake of prenatal diagnosis [17]. Women with Caucasian ethnicity were more likely to utilize prenatal diagnosis than other women. The proportion of screening was significantly lower for women of aboriginal origin [17]. It has been reported that Asian women living in the United Kingdom are less likely than white women to be offered and undergo screening for Down’s syndrome [18]. Our study showed that women, 35 years or older and with a country of birth outside of Sweden presented a 45% decreased likelihood of undergoing CUB. The lowest uptake was demonstrated by women born in Africa. Lower uptake of prenatal diagnosis in minority ethnic groups and among socioeconomically disadvantaged women, has been shown to reflect lower rates of informed choice rather than more negative attitudes towards screening [19]. Our study was not able to investigate possible effects of language barriers for pregnant women with no or little skills in Swedish to make an informed choice. In our study, country of birth had a somewhat higher impact on utilization of CUB than on utilization of CVS or AC. This might be explained by the difficulty to inform about risk evaluation and by providing pregnant women with correct information thus facilitating for making an informed choice. Pregnant women who had received counselling due to fear of childbirth utilized prenatal diagnosis to a higher degree than other women. To our knowledge, association between fear of childbirth and utilization of prenatal diagnosis has not previously been investigated. It seems likely that a higher level of anxiety could be manifested both as fear of childbirth as well as an increased concern related to the pregnancy resulting in increased number of medical procedures.

During the study period, the SPR did not include data on Non-Invasive Prenatal testing (NIPT). This method is based on analysis of cell-free fetal DNA in maternal blood [20]. In Sweden, NIPT is currently offered only in a few counties and strictly on specific indications. However, this method is accessible on the pregnant woman’s own expense.

### Methodological considerations

During the study period, the Swedish Pregnancy Register demonstrated a satisfactory coverage of pregnancies and moreover, all counties of Sweden are represented in the SPR. Data in the Swedish Medical Birth Register for 2012, demonstrate a mean maternal age, for primiparous women, of 28.4 years, and a mean BMI (all pregnant women) of 24.8 [21], and the corresponding figures in the SPR 2012, were 28.8 (years) and 24.8 (BMI), respectively. These results indicate that data in SPR are very similar to data in the Swedish Medical Register that is a compulsory health register demonstrating an almost complete coverage of pregnant women in Sweden. The validity of data in the SPR has previously been investigated and most variables demonstrate good internal validity and coverage [3]. However, the validity check also revealed that the absolute numbers of invasive prenatal diagnosis such as CVS and AC are underestimated [3]. The SPR does not include information on pregnancies with a gestational age of less than 23 weeks of gestation. Therefore, an additional limitation of this study was that different frequencies of prenatal diagnosis could not be established for this category of pregnant women that may have terminated their pregnancies.

## Conclusions

Offers of prenatal diagnostic procedures varied considerably between counties in Sweden. Maternal age, as expected, demonstrated the strongest association with the uptake of CUB, AC and CVS. Further, educational level was a strong predictor of uptake of prenatal diagnosis. These circumstances result in an unequal access of prenatal diagnostic tests for pregnant women. The intentions of the Swedish Health and Medical Services Act stating that equal care should be provided for all, was thus not fulfilled. Expecting couples should be offered the same opportunities on prenatal diagnosis nationally.

## Abbreviations

AC: Amniocentesis
ACI: Adjusted confidence interval
ANC: Antenatal Care
AOR: Adjusted odds ratio
AUDIT: Alcohol Use Disorder Identification Test
BMI: Body mass index
CI: Confidence interval
CS: Caesarean section
CUB: Combined Ultrasound and Biochemical test
CVS: Chorionic Villus Sampling
IQR: Interquartile range
MHCA: Maternal Health Care Area
MHCR: The Swedish Maternal Health Care Register
NIPT: Non-Invasive Prenatal Testing
OR: Odds ratio
SD: Standard deviation
SPR: The Swedish Pregnancy Register
